# Ethics review in compassionate use

**DOI:** 10.1186/s12916-017-0910-9

**Published:** 2017-07-24

**Authors:** Jan Borysowski, Hans-Jörg Ehni, Andrzej Górski

**Affiliations:** 1 Department of Clinical Immunology, Medical University of Warsaw, Nowogrodzka Str. 59, 02-006 Warsaw, Poland; 2 Institute of Ethics and History of Medicine, Eberhard Karls Universität, Gartenstr. 47, 72074 Tübingen, Germany; 3 Ludwik Hirszfeld Institute of Immunology and Experimental Therapy, Polish Academy of Sciences, Weigla Str. 12, 53-114 Wrocław, Poland

**Keywords:** Compassionate use, Expanded access, Research ethics committee, Institutional review board, Informed consent, Clinical trial

## Abstract

**Background:**

Compassionate use is the use of unapproved drugs outside of clinical trials. So far, compassionate use regulations have been introduced in the US, Canada, many European countries, Australia and Brazil, and treatment on a compassionate use basis may be performed in Japan and China. However, there are important differences between relevant regulations in individual countries, particularly that approval by a research ethics committee (institutional review board) is a requirement for compassionate use in some countries (e.g. the US, Spain, and Italy), but not in others (e.g. Canada, the UK, France, and Germany).

**Discussion:**

The main objective of this article is to present aspects of compassionate use that are important for the discussion of the role of research ethics committees in the review of compassionate use. These aspects include the nature of compassionate use, potential risks to patients associated with the use of drugs with unproven safety and efficacy, informed consent, physicians’ qualifications, and patient selection criteria. Our analysis indicates that the arguments for mandatory review substantially outweigh the arguments to the contrary.

**Conclusions:**

Approval by a research ethics committee should be obligatory for compassionate use. The principal argument against mandatory ethical review of compassionate use is that it is primarily a kind of treatment rather than biomedical research. Nonetheless, compassionate use is different from standard clinical care and should be subject to review by research ethics committees. First, in practice, compassionate use often involves significant research aspects. Second, it is based on unapproved drugs with unproven safety and efficacy. Obtaining informed consent from patients seeking access to unapproved drugs on a compassionate use basis may also be difficult. Other important problems include the qualifications of the physician who is to perform treatment, and patient selection criteria.

## Background

In principle, contemporary clinical practice is based on drugs that have been approved by a relevant regulatory agency for use in certain indications. Patients’ access to investigational (unapproved) therapeutics is limited to very few options, the major one being a clinical trial [1]. In recent years, increasing numbers of patients have sought access to investigational drugs on a compassionate use basis [2]. In the 1980s, the original idea underlying the development of compassionate use was to administer an investigational drug outside of a clinical trial for the direct therapeutic benefit of a patient [3]. At present, such treatment can be performed in patients with very serious, including life-threatening diseases, who cannot be satisfactorily treated with approved drugs [4–6]. Compassionate use regulations have already been introduced in the USA, Canada, most European Union countries (e.g. France, Italy, Spain, and Germany), Australia, and Brazil [7–9]. Treatment on a compassionate use basis can also be performed in Japan and China, although no specific regulations have yet been developed in these countries regarding patients’ access to unapproved therapeutics [8, 10]. There are substantial differences between the regulations adopted in individual countries. One of these pertains to the requirement for an investigational treatment to be granted approval by a research ethics committee (REC; or institutional review board, IRB, in the USA), which is mandatory in only a few countries [7].

Here we present aspects of compassionate use that are important in discussions of the role of RECs in the review of compassionate use. After detailed analysis, we found that the arguments for the involvement of a REC substantially outweigh arguments to the contrary. We conclude that REC approval should be a mandatory requirement for compassionate use.

## Discussion

### Research ethics committees

Independent review is a generally accepted ethical and legal principle for biomedical research [11]. All major international ethical and legal guidelines contain this principle, including the Declaration of Helsinki (World Medical Association) [12], the Additional Protocol to the Convention on Human Rights and Biomedicine Concerning Biomedical Research (Council of Europe) [13], the Guideline for Good Clinical Practice (International Council for Harmonisation of Technical Requirements for Pharmaceuticals for Human Use) [14], and the International Guidelines for Health-Related Research Involving Humans (Council for International Organizations of Medical Sciences with the World Health Organization) [15].

Generally, a research project cannot begin without prior approval by a REC. The REC’s primary purpose is to protect research participants, their “dignity, rights, safety, and well-being” [13], by examining whether or not the research project complies with the ethical and legal principles that regulate biomedical research. Ethical review includes, among other tasks, reviewing protocols, verifying that the researchers are appropriately qualified, determining whether there is a favorable risk:benefit ratio, reviewing documents related to the informed consent process to check whether participants are adequately informed about different aspects of the research project, and checking to see if participants have been fairly selected. Ethical review must be connected to scientific review. Scientific validity is a necessary precondition for social value, and thus for the ethical legitimacy of a research project. Without it, research participants are needlessly exposed to the risks and burdens of research. Research results that are not scientifically valid might enter medical practice, leading to patient harm and/or a waste of resources.

While the role of RECs in the review of biomedical research is commonly accepted, it is not the case with compassionate use. Currently, only a few countries, including the USA, Spain, and Italy, require REC approval for compassionate use. REC review is mandatory also in Australia, but only for programs involving groups of patients; compassionate treatment of individual patients is exempt [7].

### Should compassionate use be reviewed by research ethics committees?

To answer this question, we must discuss several key aspects of compassionate use, in particular the nature of compassionate use, the potential risks to patients associated with the use of drugs with unproven safety and efficacy, informed consent, physicians’ qualifications, and patient selection criteria.

#### Compassionate use: a unique combination of therapeutic and research aspects

The nature of compassionate use, as currently seen in practice, is complex. On the one hand, its primary purpose is to administer an investigational drug “to diagnose, monitor, or treat a patient’s disease or condition” [16], i.e., for the direct therapeutic benefit of a patient [17, 18]. The European Medicines Agency (EMA)’s Guideline on Compassionate Use of Medicinal Products, Pursuant to Article 83 of Regulation (EC) No 726/2004, developed by the Committee for Medicinal Products for Human Use (CHMP), also states that compassionate use is performed primarily for therapeutic purposes [6]. Thus, by its very nature, compassionate use is a kind of treatment, and not biomedical research. Compassionate use is similar to a clinical trial in that it involves use of an investigational drug whose efficacy and safety have not yet been shown. However, while potential benefits to enrolled patients are also considered, the primary purpose of a clinical trial is to evaluate the efficacy and safety of a drug for the benefit of future patients. Both the US Food and Drug Administration (FDA) and the EMA stress that compassionate use should be clearly distinguished from clinical trials [5, 6]. In our opinion, the general consensus that compassionate use is primarily a kind of treatment rather than biomedical research underlies the only significant argument against a requirement for independent ethical review of compassionate use: that the main role of RECs is to protect the rights of biomedical research participants [11]. Therefore, by its nature, compassionate use does not fall within the scope of interest of these committees. The main arguments for and against a requirement for independent ethical review of compassionate use are presented in Table 1.

**Table 1:** Arguments for and against the independent ethical review of compassionate use

Arguments for	Arguments against
Compassionate use often contains significant research aspects.	Compassionate use is primarily a kind of treatment.
Compassionate use is based on drugs with unproven safety and efficacy.	
Necessary to evaluate: - informed consent forms - physicians’ qualifications - patient selection criteria	

In many cases, despite not being a clinical trial, compassionate use involves (or at least should involve) some research aspects. For example, according to the Declaration of Helsinki, the most important biomedical research code [12], use of an unproven intervention in clinical practice “should subsequently be made the object of research, designed to evaluate its safety and efficacy. In all cases, new information must be recorded and, where appropriate, made publicly available” (Paragraph 37). The Declaration does not explicitly state that use of unproven intervention must be preceded by ethical review. However, the suggestion to include research aspects does seem to imply this, especially when one reads the Declaration as a whole (independent ethical review as a mandatory requirement for biomedical research involving humans is one of the most important questions discussed by the Declaration).

Walker et al. [7] recently expressed a similar view to that of the Declaration of Helsinki. They reasoned that compassionate use might be ethical, provided that it contributes to our knowledge of investigational drugs. However, they acknowledged significant shortcomings of compassionate use as a source of data on the efficacy and safety of investigational drugs. Indeed, the value of data collected during the conduct of compassionate use is limited, especially compared to that of randomized controlled trials, the contemporary gold standard of drug efficacy and safety studies [19]. Of the two major regulatory agencies, the FDA does not consider compassionate use to be a significant source of reliable safety and efficacy data; while adverse event reporting is required, this does not affect the FDA’s decisions on eventual drug approval [4, 20]. The EMA allows safety data to be collected during compassionate use programs, while at the same time stressing that compassionate use cannot replace clinical trials for investigational purposes [6].

Compassionate use may involve the treatment of individual patients (termed by the EMA as ‘compassionate use on a named patient basis’), in which the sole purpose is to obtain therapeutic benefit in a given patient, or programs involving groups of patients [5, 6], the nature of which is more complex. While compassionate use programs are generally intended to enable patients’ access to investigational drugs before formal approval, the EMA also allows them to be used to collect safety data [6]. Unlike the USA, most European countries do not require independent ethical review for compassionate use, thus in Europe even programs containing research aspects can be conducted *de facto* without REC approval. Furthermore, while the EMA guidelines explicitly allow the collection of safety data, in practice compassionate use programs are used to evaluate both the safety and the efficacy of unapproved drugs (for examples, see [21, 22]).

In recent years, compassionate use programs, including those involving large groups (often several hundreds, in some cases more than a thousand of patients), have increasingly been carried out to obtain data on the efficacy and safety of investigational drugs in “real-world” settings [23, 24]. This is possible because the inclusion and exclusion criteria used in compassionate use programs are generally less rigorous than those of randomized clinical trials [25, 26]. In our opinion, the fact that compassionate use often involves significant research aspects is the first key argument for mandatory ethical review. Such review should focus on protocols of compassionate use programs which should be subject to review by RECs similar to protocols of clinical trials. In cases where compassionate use involves no research aspects, (e.g. in the treatment of individual patients), obligatory ethical review is warranted for other reasons, to be discussed hereafter.

#### Compassionate use involves use of drugs with unproven safety and efficacy

Compassionate use programs involving large groups of patients are often carried out following the completion of Phase III clinical trials [5, 27]. However, in principle, an investigational drug may be used compassionately at any stage of its clinical development [28]. This raises questions about the safety and efficacy of investigational drugs. For example, recent analysis of 1442 investigational drugs being developed by 50 pharmaceutical firms [29] found that the overall probability of a drug in clinical testing eventually being approved was as low as 11.83%. Phase transition probability was estimated at 59.52% (Phase I–II), 35.52% (Phase II–III), 61.95% (Phase III–New Drug Application/Biologic License Application (NDA/BLA) submission, and 90.35% (NDA/BLA submission-NDA/BLA approval). Thus, even at later stages of clinical trials, or the regulatory review stage, the percentage of drugs that fail is considerable.

While the authors did not report the percentage of drug attrition in individual phases caused by safety or efficacy concerns, another study from this group showed that safety concerns (including toxicity) accounted for 20.5%, and lack of efficacy for 35.3% of failures [30]. Thus, combined safety and efficacy concerns may account for more than half of investigational drug attritions. Some authors suggest that the percentage of investigational drugs abandoned at different clinical trial stages because of safety concerns might be even higher [31]. Another study of 640 investigational drugs in late-stage clinical development revealed that 54% failed during or after pivotal clinical trials; most of these failed because of inadequate efficacy (57%) or safety concerns (17%) [32].

Data presented here show that any use of investigational drugs (even those from late-stage clinical trials or undergoing regulatory review) may be associated with significant safety and/or efficacy concerns. This is the second key argument for compassionate use to be subject to mandatory ethical review; an important role of a REC should be to evaluate whether the potential benefits of administering an investigational treatment of unproven safety and efficacy justifies exposing a patient to the risks associated with its use. In many cases such evaluation will be difficult; for example, whether it is ethical to treat a patient with a life-threatening disease with a drug that might not only be ineffective, but which might also deteriorate their condition. Ethical dilemmas associated with patients’ potential access to unapproved drugs are complex [33], and it is beyond the scope of this article to discuss these. Nonetheless, the moral and practical importance of these dilemmas seems to justify the involvement of RECs in the review of compassionate use.

#### Importance of informed consent in compassionate use

By definition, and as clearly specified in both FDA regulations and EMA guidelines [5, 6], compassionate use is for patients with very serious, including potentially life-threatening diseases, who cannot be satisfactorily treated with approved drugs. Data on the efficacy and safety of drugs at early stages of development are limited, and patients may have no access to important data, nor the knowledge necessary to evaluate them [34]. Therefore, patients who wish to be treated with an unapproved drug may overestimate its potential benefits and have a poor understanding of the possible risks. Information about the nature and likelihood of potential ill effects is an essential condition for the adequate comprehension of risk [35]. Therefore, as pointed out by other authors [27, 34] such patients are particularly vulnerable.

In the context of compassionate use, two specific situations can arise in which a patient’s vulnerability might be associated with potential abuse. The first is in cases where a treating physician is also a researcher. While involving patients in biomedical research is ethically permissible (see for example the Declaration of Helsinki, paragraph 14 [12]), there may be a conflict of interest when research involving a patient takes precedence over the clinical care of this patient [36]. Since compassionate use often combines therapeutic and research aspects, a conflict of interest might arise, for example, from a physician’s desire to “pioneer” the use of a novel intervention without paying sufficient attention to the patient’s medical needs [7].

The second situation that harbors potential for abuse is associated with the commercial sales interests of drug manufacturers. The possibility exists that these manufacturers might use compassionate use programs to distribute investigational drugs, thus generating increased demand for the drug following its eventual formal approval. Compassionate use programs may also provide patients with continued access to a drug that is eventually not approved. Positive results obtained in compassionate use programs can also generate unwarranted beliefs regarding the efficacy and safety of investigational drugs, and in some cases this might be used to try to fast-track the drug’s formal approval [7]. There is a risk, therefore, that the commercial interests of drug manufacturers might influence decisions on which drugs, and which patients, are included in compassionate use programs.

In some countries, drugs for compassionate use must be dispensed free of charge [17]. Consequently, it may be problematic when the free supply of a drug ceases following its introduction into the national market and/or the national reimbursement list. This situation, together with a very advanced stage of clinical development, might result in a drug’s accelerated, “forced” admission to the hospital formulary, not only for the concerned patient, but also for others with the same disease.

A properly prepared informed consent form is a basic safeguard to prevent potential abuse, and to ensure that a treatment is compassionately used primarily for the patient’s benefit. It is beyond the scope of this article to discuss the specific information that should be included on this form, but it should contain adequate information about the potential benefits and risks associated with the use of drugs with unproven safety and efficacy. While it is commonly accepted that informed consent should be required for compassionate use [4, 17, 27], independent review would ensure that informed consent forms really fulfill their purpose [4]. In practice, RECs have extensive experience of reviewing informed consent forms, and although they are principally involved in the evaluation of those used in biomedical research, the overall objective of the consent form in compassionate use is the same: to present a patient with all the risk/benefit information they need to make a truly informed choice about participation in a program. Therefore, consent forms to be used in compassionate use, especially in programs involving research aspects, should be reviewed by RECs. Where compassionate use does not involve research (e.g. in the treatment of individual patients) an alternative solution could be to codify consent form standards. Physicians wanting to administer a drug on a compassionate use basis could then be held to those standards.

#### Physician’s qualifications

Previous reviews have not discussed the importance of the qualifications held by a physician who gives compassionate treatment. These qualifications should be particularly high, for several reasons. First, patients who wish to be treated with investigational drugs on a compassionate use basis have very serious medical conditions. Second, any use of drugs with unproven safety and efficacy is associated with a high risk of side effects or other complications. Furthermore, when considering a patient for compassionate use, the physician should take into account not only medical aspects, but also the complex ethical dilemmas inherently connected with the use of unapproved drugs. Therefore, the physician should possess not only high professional qualifications, but also have some background of ethical training, or at least have an awareness of the ethical aspects of compassionate use. In this regard, note that the Declaration of Helsinki states that, “medical research involving human subjects must be conducted only by individuals with the appropriate ethics and scientific education, training and qualifications” (General Principles section, paragraph 12 [12]). The Good Clinical Practice standards [14] also state that the qualifications of the principal investigator should be evaluated during the independent ethical review of a clinical trial proposal (Institutional Review Board/Independent Ethical Committee section, paragraphs 3.1.2 and 3.1.3).

For these reasons, in our view, RECs should evaluate the qualifications of physicians giving compassionate treatments as part of their review of compassionate use requests, as is the case with clinical trials. Physicians’ qualifications might also be evaluated by an administrative body within hospitals, similar to the approval of physicians for privileges to perform certain procedures.

#### Fair patients selection is essential for ethical compassionate use

Since it involves the use of investigational drugs, compassionate treatment is not considered standard clinical care. Consequently, problems of inequity may arise in terms of patients’ access to compassionate use programs. First, manufacturers have no legal obligation to expand access to investigational drugs outside clinical trials [37]. Second, not all patients have equal access to information about these drugs [34]. Rules regulating the use of unapproved drugs may also include strict medical indications that, in some cases, might be ambiguous [7]. Furthermore, patients’ access to compassionate use programs is, to some extent, dependent on economic and social factors, including the ability to exploit social media [2]. Fair patient selection is therefore an important ethical challenge in compassionate use [28]. Access to unapproved drugs should depend only on medical criteria (most often that a patient with a serious or life-threatening disease cannot be satisfactorily treated with any approved drug). Ensuring fair selection of patients is another important role for RECs, who can draw on their extensive experience of evaluating fair clinical trial enrollment [36]. These experiences would be particularly relevant to compassionate use programs involving research.

#### Independent ethical review of compassionate use: practical aspects

Lengthy review processes might cause reluctance to obtain REC approval in compassionate use cases [27]. This problem has been recognized by the FDA, which requires IRB approval for all kinds of compassionate use, but admits that in some cases this requirement could deter access of patients to unapproved drugs [20]. Discussing how the Independent Ethics Committee of the University Hospital of Bologna deals with compassionate use requests, Montanaro et al. [38] recently suggested that such requests might undergo a fast-track review process to avoid delays. While this committee reviews clinical trial proposals during monthly plenary meetings, a specific working group is responsible for making decisions to authorize compassionate use requests, usually within 72 hours. Favorable opinions of the group may be executed immediately.

A similar solution could be adopted by other RECs to avoid considerable delays in the conduct of compassionate treatment. Regretfully, to the best of our knowledge, this is the only report on how RECs deal with compassionate use requests. Importantly, in emergency situations when any delay can harm a patient (see, for example, [39]), treatment with an unapproved drug could be initiated quickly, without ethical review, with the REC being notified later. This is allowed, for example, by the FDA regulations [20].

Currently, in those countries that require it, independent ethical review of compassionate use is performed by RECs, which so far have been largely involved in the review of biomedical research [5, 27]. Darrow et al. [34] suggest that, in future, multicenter committees could be established to review only compassionate use requests. This might ensure more effective ethical oversight of compassionate use. Another promising option is to establish committees like the recently reported Compassionate Use Advisory Committee (COMPAC). This independent committee, established at New York University Langone Medical Center at the request of a major pharmaceutical company that offers compassionate use programs, comprises members including physicians, bioethicists, patients, and patient advocates. COMPAC (and possibly other similar committees in future) may also contribute to setting high ethical standards of compassionate use by ensuring fair patient selection [28].

## Conclusions

The principal argument against a requirement for the independent ethical review of compassionate use is that it is, in theory, primarily a kind of treatment rather than biomedical research. However, this assumption fails to take into account that, in practice, compassionate use may also involve significant research aspects, especially in programs involving groups of patients. This is the first key argument for the mandatory ethical review of compassionate use.

The second key argument is that compassionate treatment is based on drugs with unproven safety and efficacy, which require a careful evaluation of the risk:benefit ratio. This makes obtaining informed patient consent particularly difficult.

Thus, despite the fact that compassionate use is not equivalent to biomedical research, patients who are to be treated with unapproved drugs on a compassionate use basis are in much greater need of specific protection than those in standard clinical care. RECs should be involved in safeguarding this protection. The independent ethical review of compassionate use should consider protocols (in cases of programs involving groups of patients), available drug safety and efficacy evidence, informed consent forms, medical justifications for using an unapproved drug, and the administering physician’s qualifications.

## Abbreviations

BLA: biologic license application
CFR: Code of Federal Regulations
CHMP: Committee for Medicinal Products for Human Use
COMPAC: Compassionate Use Advisory Committee
EMA: European Medicines Agency
FDA: Federal Drug Administration
IEC: independent ethical committee
IND: investigational new drug
IRB: institutional review board
NDA: new drug application
REC: research ethics committee
